# Surgical Selection of Unstable Intertrochanteric Fractures: PFNA Combined with or without Cerclage Cable

**DOI:** 10.1155/2021/8875370

**Published:** 2021-02-08

**Authors:** Chaoqing Huang, Xing Wu

**Affiliations:** Department of Orthopedics, Shanghai Tenth People's Hospital, School of Medicine, Tongji University, Shanghai 200072, China

## Abstract

Due to the instability of unstable intertrochanteric fractures, the selection of a suitable internal fixation has always been a challenge for orthopedic surgeons. This study is aimed at comparing the clinical efficacy of PFNA combined with cerclage cable and without cerclage cable and finally recommend a stable internal fixation method to provide the basis for clinical therapy. From January 2014 to January 2018, we retrospectively analyzed all cases of unstable intertrochanteric fractures who received treatment in the Orthopedics Department of our hospital and finally screened 120 cases, 51 of whom were treated with cerclage cable, 69 without cerclage cable. The follow-up period was one year. HHS, BI, and RUSH scores were given within the specified time. We divided the patients into the PFNA+cable (PFNA combined with cerclage cable) group and the PFNA group. The time of fracture healing and weight-bearing in the PFNA+cable group was shorter than that in the PFNA group. With regard to HHS, BI, and RUSH, the PFNA+cable group was higher than the PFNA group at 1 month, 3 months, 6 months, and 12 months after operation. For HHS rating, the PFNA+cable group has a higher excellent rate than the PFNA group, which was 96.1% and 84.1%, respectively. All the results mentioned above were statistically significant. Compared with the group without cerclage cable, the application of cerclage cable can reduce the incidence of complications. From the comparison between the two groups, it can be seen that the surgical method of PFNA combined with cerclage cable can not only help to improve the stability of fracture reduction, shorten the time of fracture healing and postoperative weight-bearing, and significantly improve patients' self-care ability but also reduce the incidence of postoperative complications. Therefore, we think PFNA combined with cerclage cable is a good choice.

## 1. Introduction

The most common fractures in femur near-end bone fracture are femur neck, intertrochanteric, and subtrochanteric fractures, accounting for approximately 45%, 45%, and 10%, respectively [1]. Among them, intertrochanteric fractures are more common in the elderly; the main reason is that the elderly have poor physical conditions, often accompanied by osteoporosis, cardiovascular and cerebrovascular diseases, and other underlying diseases, often caused by low-energy injuries; therefore, the disability rate and death rate of intertrochanteric fractures are higher [2–4]. In the wake of developments in science and technology, the increasing number of vehicles, and the growing population of the elderly, the incidence of intertrochanteric fractures has increased rapidly. Therefore, the treatment and postoperative functional recovery of intertrochanteric fractures has become hot topics for orthopedic surgeons in recent years.

In recent years, with the deepening of orthopedic doctors' understanding and research on intertrochanteric fractures, according to the characteristics of intertrochanteric fractures such as anatomy and prognosis, more and more classification systems of intertrochanteric fractures have been developed, among which the most commonly used is the AO/OTA classification system. According to the AO classification principle, intertrochanteric fractures were subdivided into 31A1, 31A2, and 31A3 [5]. Among them, unstable intertrochanteric fractures include 31A2.2, 31A2.3, 31A3.1, 31A3.2, and 31A3.3. Currently, conservative treatment and surgical treatment are the main treatment strategies of unstable intertrochanteric fractures. As a result of gypsum fixation is easy to cause pulmonary infection, venous thrombosis, malnutrition, bedsore, urinary system infection, joint stiffness, and other complications, so choose a surgical remedy is the optimal therapeutic regimen. PFNA, DHS, PFN, Intertan, and Gamma are the most common fixation methods for unstable intertrochanteric fractures [6, 7]. Among them, PFNA has the advantages of small incision, less bleeding, and firm fixation, but the operation of PFNA is highly required of skill [8]. We need time, skills, and more tools during surgery. In addition, when closed reduction is difficult, open reduction should be performed, but in order to transfer the weight of human body through aligned cataclasis debris and avoid cataclasis displacement during operation, it is necessary to use various reduction techniques used for auxiliary surgical treatment, such as cerclage cable, to restore the function of abductor and repair the trochanteric fracture. In complex proximal femoral fractures, although the use of cerclage cable is still controversial or ambiguous, its potential application value has been advocated [9]. However, with regard to the surgical cure for unstable intertrochanteric fractures, few researches have proposed to apply cerclage cable for treatment. Therefore, this research has a view to compare the clinical efficacy of PFNA with or without cerclage cable, so as to recommend a remedy scenario that can provide stable fixation for unstable intertrochanteric fractures and provide basis for clinical therapy.

### 1.1. Surgical Technique

**Figure 1:**
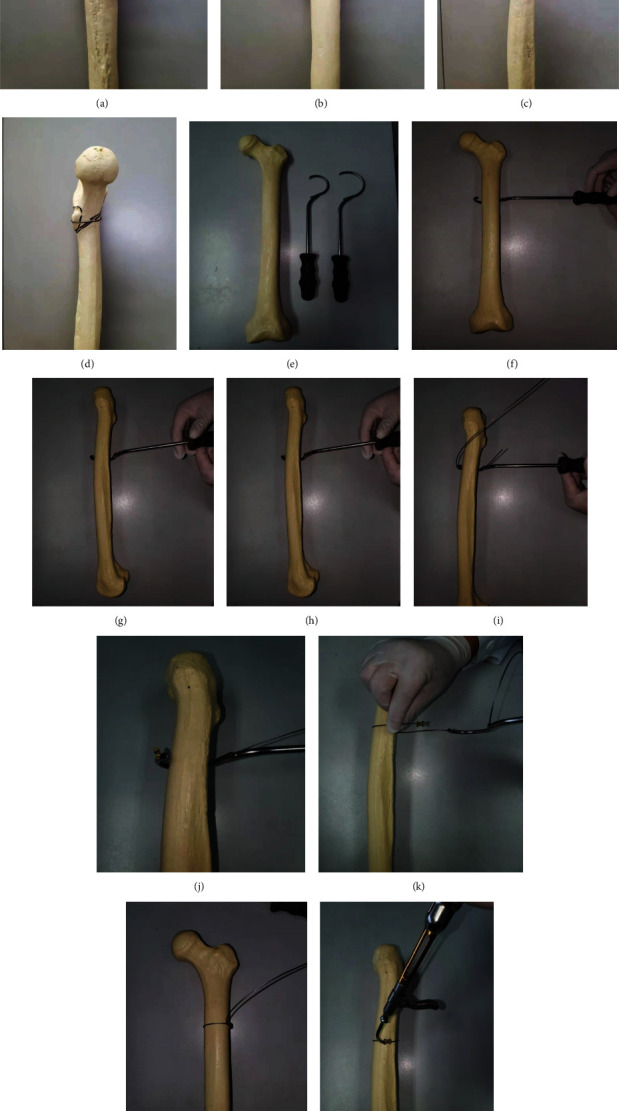
The model picture after PFNA+cable operation and the detailed surgical procedure of cerclage cable.

**Figure 2:**
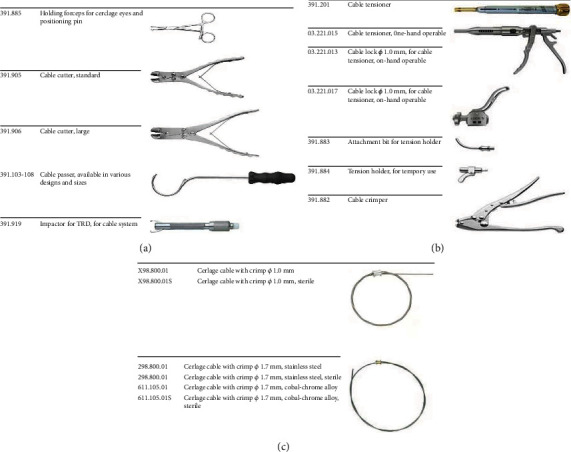
The surgical instruments used in our cerclage cable operation.

After successful general anesthesia, patients lay supine on a special orthopedic surgical traction table, traction and fixation of both lower extremities, straightening and internal rotation of affected limbs by 15°, then moderate abduction of healthy limbs. After successful closed reduction, guide wire was inserted and the hollow drill was used to enlarge the medullary cavity; then, the PFNA intramedullary nail of appropriate length was inserted. The spiral blade was placed with the help of guide wire and guide sleeve; then, the distal interlocking nail of appropriate length was installed. Under the premise of closed reduction failure, it is particularly important to implement limited open reduction. With the assistance of X-ray fluoroscopy, fluoroscopy was used for the fracture site. If the reduction is satisfactory, the proximal femur intramedullary nail is placed and fixed routinely. Usually, there are flexion, adduction, and external rotation deformities at the proximal end of the fracture, and closed reduction is difficult to meet the reduction requirements. In this case, a small incision of 5 to 6 cm is made on the outer thigh at the level of the fracture line for limited open reduction. After the deep fascia was incised, the lateral femoral muscle was bluntly separated until the fractured end was touched, and the pointed reduction forceps were used for reduction under fluoroscopic guidance. The pointed reduction forceps are clamped in the front and rear directions, and a slight external rotation of the distal end of the fracture can correct the rotational displacement. The adduction deformity is usually corrected with the tightening of the pointed reduction forceps. Then, select the appropriate cable passer. The size and shape of the cable passer depend upon the circumference of the bone and access to the site. Select a cable passer that will allow the instrument to pass around the bone without causing significant damage to soft tissues or excessive stripping of the periosteum. Pass the cable passer around the bone. Thread the free end of the cable into the end-hole of the cable passer until the cable exits through the shaft hole. Remove the cable passer leaving the cable wrapped around the bone. Insert the end of the cable through the free hole of the crimp, and place the crimp in the desired position on the bone. When placing the crimp, ensure that it is covered by soft tissue and securely anchored in the bone. The four points on the underside of the crimp must contact the bone, and the smooth side must face upwards. Mount the temporary tension holder and the attachment bit on the cable tensioner. To enable the cerclage cable to be inserted into the cable tensioner, turn the fluted knob at the end of the tensioner counterclockwise as far as possible. Insert the cerclage cable into the cable tensioner, and advance the attachment bit up to the crimp. Turn the fluted knob on the cable tensioner until the desired tension is reached. The tension is shown by the markings on the tensioner. To temporarily fix a cerclage cable, the cable tensioner can be removed without causing loss of tension thanks to the temporary tension holder. Pull back the lever of the cam lock on the temporary tension holder, and loosen and remove the cable tensioner. Using this procedure, any cerclage cable can be retensioned and/or repositioned before definitive fixation. When the desired cable tension is reached, the cerclage cable can be secured with the crimp. Place the jaws of the cable crimper on the crimp, ensuring that the crimp is centred and is correctly held in the crimper jaws. Pull the inner start lever first, then squeeze the outer handles to complete crimping. The toothed mechanism of the cable crimper establishes the appropriate compression pressure for securing the crimp. When the crimp—and thus the cerclage cable—is secured, turn the fluted knob on the cable tensioner as far as possible, and remove the tensioner. If the temporary tension holders are wed, push the lever of the cam lock forward, and pull the holder off the cable. Cut the loose end of the cable using the cable cutter. Position the cutting jaws very close to the crimp, and make the cut in one action to produce a clean cut. Ensure that the adjacent cerclage cables do not get damaged (see Figures 1(e)–1(m)). Figures 1(a)–1(d) show the model picture after PFNA+cable operation.  Figure 2 shows the surgical instruments used in our cerclage cable operation.

## 2. Materials and Methods

### 2.1. Ethical Statement

Inform the patients and/or family members of the purpose and nature of this study, and sign a written informed consent form after obtaining their consent. Our clinical study complies with the provisions of the ethics committee of Shanghai Tenth People's Hospital, School of Medicine, Tongji University.

### 2.2. Patients

From January 2014 to January 2018, all sufferers with unstable intertrochanteric fractures who received treatment in the Department of Orthopedics, Shanghai Tenth People's Hospital, School of Medicine, Tongji University, and the final selected cases were all elderly. The inclusion criteria are as follows: age of these team members is more than 60 years; according to the AO/OTA classification system and X-ray examination, patients who were diagnosed with unstable intertrochanteric fractures; and continuous follow-up for one year. After screening, we finally screened 120 cases, 51 of whom were treated with cerclage cable, 69 without cerclage cable.

### 2.3. Grouping and Treatment

Patients were divided into the PFNA group and the PFNA combined with cerclage cable group. All patients were examined in detail before operation and were treated with anti-infection, detumescence, pain relief, and other symptomatic treatment. Patients in the PFNA group were treated with PFNA operation and patients in the PFNA combined with cerclage cable group received PFNA operation combined with cerclage cable. After operation, we actively prevent complications such as pulmonary infection, venous thrombosis, bedsore, urinary system infection, and osteoporosis, urge patients to actively carry out rehabilitation exercise to prevent joint stiffness, and promote bone reconstruction.

### 2.4. Follow-Up and Observation Indexes

Postoperative observation indexes include operation time, intraoperative blood loss, weight-bearing time, and fracture healing time. The evaluation criterion of articulatio coxae function was Harris hip score (HHS) [10]. Activities of daily living (ADL) were detected by Barthel Index (BI) [11]. In intertrochanteric fractures, Radiographic Union Scale for Hip (RUSH) was used for iconography evaluation of fracture healing [12]. HHS and BI were first evaluated before operation, and then, HHS, BI, and RUSH were evaluated at 1, 3, 6, and 12 months after operation, respectively.

### 2.5. Statistical Analysis

The statistical analysis tool we used in this study was SPSS 21.0 software, and the expression form of measurement data is mean ± standard deviation. The *t*-test was used for the comparison of measurement data, and the chi-square test was used for the comparison of enumeration data.

## 3. Results

**Figure 3:**
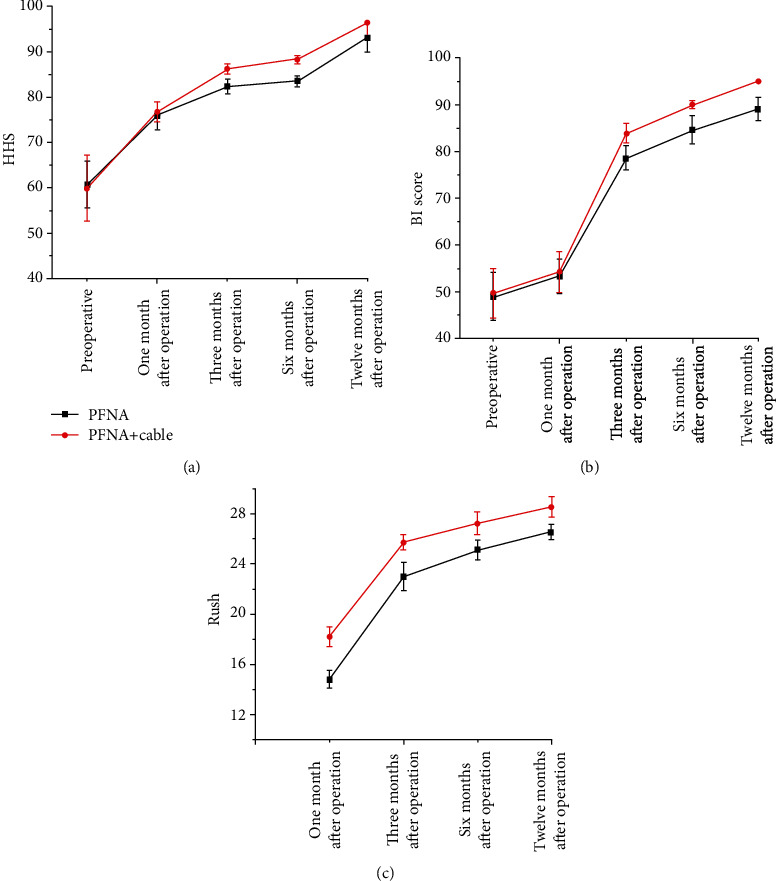
Compare the trends of HHS, BI score, and RUSH between the two groups Figure notes: (a) HHS; (b) BI; (c) RUSH.

**Figure 4:**
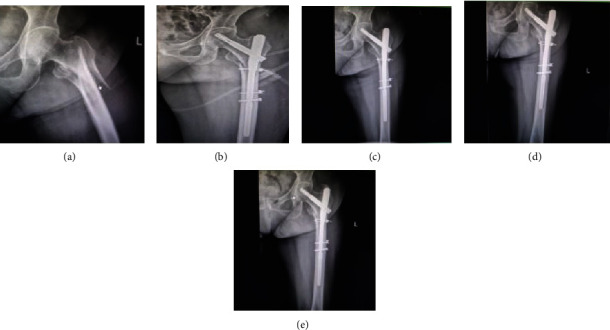
Preoperative and postoperative radiographic data of a patient with reverse oblique intertrochanteric fracture treated with PFNA+cable. Figure notes: (a) preoperative; (b) one month after operation; (c) three months after operation; (d) six months after operation; (e) twelve months after operation.

**Figure 5:**
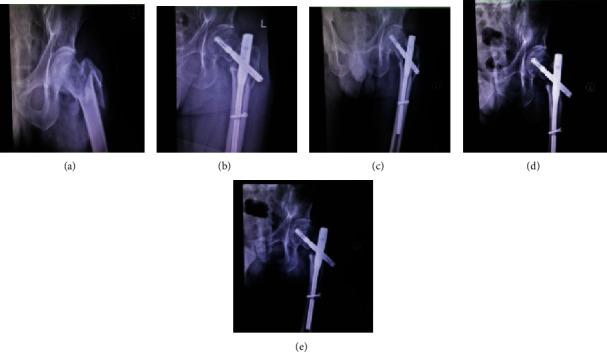
Preoperative and postoperative radiographic data of a patient with reverse oblique intertrochanteric fracture treated with PFNA. Figure notes: (a) preoperative; (b) one month after operation; (c) three months after operation; (d) six months after operation; (e) twelve months after operation.

All sufferers were fixed with PFNA (Synthes®, Oberdorf, Switzerland). The follow-up time of all invalids was one year; then, the baseline information and follow-up data of all invalids were recorded in detail. In this study, 120 cases of unstable intertrochanteric fractures can be divided into 51 cases treated with cerclage cable and 69 cases not treated with cerclage cable. The age distribution of the two groups has no statistical sense (*p* = 0.306), and the mean age of sufferers in the PFNA+cable group and the PFNA group was 83.0 ± 10.6 years and 84.7 ± 7.5 years, respectively. Also, the BMI (*p* = 0.139) and gender (*p* = 0.941) of the two groups have no statistical sense. In terms of hypertension (*p* = 0.28), diabetes (*p* = 0.822), heart disease (*p* = 0.243), and other diseases (*p* = 0.119), the difference between the two groups is minimal. Little difference has been found between the two groups in ASA grading and AO classification; the specific statistical results are as follows: ASA II (*p* = 0.356), ASA III (*p* = 0.28), and ASA IV (*p* = 0.879); 31A2.2 (*p* = 0.86), 31A2.3 (*p* = 0.211), 31A3.1 (*p* = 0.867), 31A3.2 (*p* = 0.133), and 31A3.3 (*p* = 0.82) (see Table 1). The difference of intraoperative blood loss (*p* = 0.214) and operation time (*p* = 0.064) between the two groups was very small. The mean weight-bearing time and mean fracture healing time of the PFNA+cable group were 2.94 ± 0.27 months and 3.36 ± 0.23 months, respectively, while those of the PFNA group were 3.73 ± 0.71 months and 4.34 ± 0.22 months. The weight-bearing time (*p* = 0.0001) and fracture healing time (*p* = 0.0001) of the PFNA+cable group were significantly shorter than those of the PFNA group. After follow-up, the mean Harris hip scores in the PFNA+cable group were 59.9 ± 7.3, 76.7 ± 2.2, 86.2 ± 1.1, 88.2 ± 0.8, and 96.4 ± 2.9 at preoperative, 1 month after operation, 3 months after operation, 6 months after operation, and 12 months after operation, while in the PFNA group were 60.7 ± 5.2, 75.9 ± 2.8, 82.3 ± 1.6, 83.5 ± 1.2, and 93.1 ± 3.2. After statistical analysis, there was no statistical significance in HHS at preoperative (*p* = 0.485) and 1 month after operation (*p* = 0.075), but it was statistically significant at 3 months (*p* = 0.0001), 6 months (*p* = 0.0001), and 12 months (*p* = 0.0001) after operation. Analysis results have shown that the PFNA+cable group was superior to the PFNA group in the recovery of hip joint function from 3 months to 12 months after operation. In other words, the use of cerclage cable can maintain fracture reduction and improve the stability of fracture reduction. The mean BI scores in the PFNA+cable group were 49.7 ± 5.3, 54.2 ± 4.4, 83.8 ± 2.1, 89.9 ± 0.7, and 95.0 ± 0.0 at preoperative, 1 month after operation, 3 months after operation, 6 months after operation, and 12 months after operation, while in the PFNA group were 48.9 ± 5.2, 53.3 ± 3.7, 78.6 ± 2.6, 84.6 ± 3.1, and 89.1 ± 2.5. With regard to the comparison of patients' ADL, there was no statistical significance in BI at preoperative (*p* = 0.410) and 1 month after operation (*p* = 0.227), but it was statistically significant at 3 months (*p* = 0.0001), 6 months (*p* = 0.0001), and 12 months (*p* = 0.0001) after operation. From data comparison, we can see that the ADL of the PFNA+cable group is better than that of the PFNA group from 3 months to 12 months after operation. Therefore, the use of cerclage cable can improve patients' ADL. During the follow-up, we compared the mean RUSH scores of the two groups; the mean RUSH scores in the PFNA+cable group were 18.2 ± 0.8, 25.7 ± 0.6, 27.2 ± 0.9, and 28.5 ± 0.8 at 1, 3, 6, and 12 months after operation, respectively, while in the PFNA group were 14.8 ± 0.7, 23.0 ± 1.1, 25.1 ± 0.8, and 26.5 ± 0.6. In terms of imaging score, after statistical analysis, it was found that the mean RUSH scores of the two groups were statistically significant at 1 month (*p* = 0.0001), 3 months (*p* = 0.0001), 6 months (*p* = 0.0001), and 12 months (*p* = 0.0001) after operation. The results show that the PFNA+cable group had better fracture healing than the PFNA group (see Table 2). During the last follow-up period, we compared the HHS rating between the two groups. It can be seen that the proportion of excellent in the PFNA+cable group accounted for 96.1%, while the proportion of excellent in the PFNA group accounted for 84.1%. The results of excellent rate show no remarkable difference between the two groups (*p* = 0.036). Therefore, we can also infer that the PFNA+cable group was superior to the PFNA group in postoperative hip joint function recovery effect (see Table 3). Through the HHS trend chart, we can see that the HHS of the two groups are increasing with the passage of time, but the PFNA+cable group had higher HHS than the PFNA group. The HHS growth trend of the two groups is flat from 3 months to 6 months after operation, but the HHS of the two groups is gradually increasing from 6 months to 12 months after operation. We can see from the figure that the hip joint function of both groups has improved over time, but the recovery rate of hip joint function in the PFNA+cable group was faster than that in the PFNA group. The hip joint function of the two groups was close to normal from 3 months to 6 months after operation, so function recovery of articulatio coxae is slow, and the hip joint function continues to return to normal from 6 months to 12 months after operation (see Figure 3(a)). From the comparison of BI scores, we can see that BI scores of the two groups increased over time. However, BI scores of the PFNA+cable group increased faster than that of the PFNA group. The growth rate of BI scores of the two groups was the fastest from 1 month to 3 months after operation, and the growth rate slowed down from 3 months to 6 months after operation. Therefore, it can be inferred that the PFNA+cable group had better ADL than the PFNA group, and patients in both groups had the fastest improvement in ADL from 1 to 3 months after operation and then gradually recover to normal ADL from 3 to 12 months after operation (see Figure 3(b)). In terms of the RUSH score, the fracture healed continuously over time. The fracture healing rate was the fastest in 1 to 3 months after operation, and then, the fracture healing rate slowed down and gradually reached the fracture healing. As far as the mean RUSH score is concerned, it can be clearly seen from the figure that the mean RUSH score of the PFNA+cable group is higher than that of the PFNA group, so we speculate that the PFNA+cable group will heal faster than the other group (see Figure 3(c)). During the follow-up, no patient died, and many postoperative complications occurred; the specific complications are as follows: two cases of superficial wound infection occurred in the PFNA group, while only one case occurred in the PFNA+cable group, all patients received symptomatic treatment with antibiotics, and the wounds healed well after treatment; three patients developed deep infection in the PFNA group, compared with only two in the PFNA+cable group. All patients received debridement and antibiotic treatment, of which two patients in the PFNA group needed VSD treatment to promote wound healing, while only one patient in the PFNA+cable group needed VSD treatment, and finally, the wounds of all patients with deep infection healed well; in the PFNA group, one case had fracture nonunion, two cases had screw penetration, and one case had screw cut-out; all the patients mentioned above underwent secondary surgery, but the above phenomenon did not occur in the PFNA+cable group (see Table 4). From the comparison of Figures 4 and 5, we can see that the fracture of the patients fixed with PFNA+cable has partially healed at one month after operation, but the patients fixed with PFNA still do not start to heal. At twelve months after operation, the fracture of the patients fixed with PFNA+cable has healed, while the patients fixed with PFNA have fracture nonunion. Finally, we choose total hip replacement to treat fracture nonunion.

## 4. Discussion

Our results showed that patients without cerclage cable were prone to fracture displacement during later follow-up, while patients with cerclage cable were not prone to fracture displacement during later follow-up. Therefore, we believe that take advantage of cerclage cable can keep the fracture in good anatomical reduction or close to anatomical reduction. Only on the premise of maintaining good reduction can we avoid the displacement of fracture fragments or even bone split in the process of nail placement, and the use of cerclage cable can stabilize the aligned fracture fragments, and most human body weight can be transferred through aligned bone fragments, so that the whole fixation can achieve a more stable effect.

In the selection of internal fixation before operation, due to the instability of unstable intertrochanteric fractures, it is particularly important to select the appropriate internal fixation, which can not only achieve stable anatomical reduction but also maintain reduction until fracture healing, so as to shorten the weight-bearing time of patients and reduce the incidence of postoperative complications. During the surgical procedure, when closed reduction cannot be successful, we recommend open reduction and use cerclage cable after open reduction to achieve a more stable fracture reduction effect. Afsari et al. believe that proper use of cerclage cable for clamp-assisted reduction and intramedullary nail fixation can achieve a good reduction effect and high fracture healing rate [13]. Kulkarni et al. proposed that cerclage cable can improve the fixation strength of intramedullary nail and thus help to reduce the incidence of surgical complications [14]. Apivatthakakul et al. believe that cerclage cable can help reduce and maintain refractory intertrochanteric fractures [15]. Kilinc et al. proposed that open reduction and the use of cerclage cable have no adverse effect on fracture healing [16]. It has been reported that the effect of adding minimally invasive cerclage cable to the subtrochanteric fracture is similar to that of the reverse intertrochanteric fracture [13]. Compared with that without cerclage cable, the reoperation rate and reduction quality with cerclage cable are improved, and the displacement of fracture is also reduced [17, 18]. In clinical practice, in order to obtain the correct nail insertion point to achieve the anatomical reduction or close to the anatomical reduction, under the premise of the correct nail placement process, some patients must undergo open reduction, which is due to the role of abductor muscle that easily lead to distal fracture and proximal fracture displacement [19, 20]. Due to unsuccessful closed reduction, we suggest to use the cerclage cable after open reduction, because the use of cerclage cable can not only maintain the initial stability of the fracture site but also reduce the abduction of the proximal fracture. In early weight-bearing, the cerclage cable can stabilize the aligned bone fragments, so that the weight of human body can be transferred through the aligned bone fragments, which is not only conducive to maintaining fracture reduction but also reduces the incidence of postoperative complications. After statistical analysis of clinical data, we found that the PFNA+cable group had shorter weight-bearing time than the PFNA group, which was mainly due to the stable reduction of fracture fragments provided by the cerclage cable, thus shortening the weight-bearing time after operation. According to a biomechanical report, cerclage cable not only improves the probability of successful bone synthesis in complex fractures but also provides important posterior medial support for unstable intertrochanteric fractures [21]. For young patients, when closed reduction is difficult during surgical dealings with intertrochanteric fractures, take advantage of steel cable is a good settlement for this problem; it is not only easy to drill and place nails, reduces the occurrence of intraoperative complications, but also conducive to the protection of fracture reduction [22]. It has been proposed that the use of cerclage cable destroys not only the blood vessels at the fracture site but also the blood supply for fractures, which leads to nonunion [23]. Someone in a study of an animal model without fracture found that the use of cerclage cable could protect the supportive blood vessels at the fracture site [24]. After follow-up, it was found that all the patients who had been treated with cerclage cable were completely healed, so we thought that cerclage cable had no harmful effect on fracture healing. Some people believe that the normal fracture healing time will not be affected by the use of steel cable [25]. Intramedullary nail (IMN) is the preferred treatment for subtrochanteric fractures, especially unstable intertrochanteric fractures [26–32]. PFNA has been shown to retard rotation and medial cortical collapse by biomechanics, mainly because the spiral blade can compress cancellous bone to increase its stability. Biomechanical tests have also shown that in osteoporotic bone, the cut-out rate of PFNA spiral blades is lower than commonly used screw systems [33, 34]. Therefore, we use PFNA to treat patients with unstable intertrochanteric fractures. Cerclage cable can keep fracture reduction longer [35]. It has been suggested that the use of cerclage cable requires a larger incision, which also causes greater damage to the soft tissue and periosteal circulation [36]. Some studies have suggested that the periosteum is dissected with multiple musculoperiosteal vessels nourishing and the direction of these musculoperiosteal vessels is circumferential rather than longitudinal [37]. Some studies have shown that the periosteal circulation damage caused by cerclage cable is negligible in oblique or spiral intertrochanteric fractures [38]. In biomechanics, intramedullary fixation has more obvious advantages than extramedullary fixation [39]. Many clinical studies have proposed that intramedullary fixation can not only decrease the accidence of internal fixation failure but also speed up fracture healing, which can make patients bear weight at an early stage. Meanwhile, complications such as venous thrombosis, bedsore, respiratory tract, and urinary tract infection are also significantly reduced [40–42].

We think that the application of cerclage wire fixation has the following advantages: (1) It is conducive to fracture reduction and maintenance of reduction, so it is easy to establish the correct intramedullary nail tunnel and insert the intramedullary nail. (2) It is beneficial to increase the contact area of cortical bone at fracture end, so as to accelerate fracture healing, quicken the healing speed of fracture, and decrease the accidence of fracture nonunion. (3) It is beneficial to improve the intensity of internal fixation. Patients can walk down early, especially for elderly patients. (4) It facilitates stress transfer between bone and internal fixation and reduces complications.

Our suggestions for using the cerclage cable are as follows: (1) The placement position of the cerclage wire during the operation needs to be accurately judged, so as to avoid being affected when placing screws in the head and neck. (2) The cerclage cable is inserted with the assistance of the cerclage cable guide, without excessive stripping of soft tissue, especially medial soft tissue, so as not to affect fracture healing and damage blood vessels.

## 5. Conclusions

In summary, through this clinical study, we believe that the application of cerclage cable is not only conducive to fracture reduction but also conducive to the maintenance of reduction. After analysis, we will summarize the use of cerclage cable as follows: first, it can stabilize fracture reduction until fracture healing; second, it can shorten weight-bearing time and speed up fracture healing; third, it can significantly improve patients' self-care ability; finally, it can reduce the occurrence of complications. Therefore, in the surgical dealings with unstable intertrochanteric fractures, since take advantage of cerclage cable has a better clinical effect than nonuse cerclage cable, we recommend that orthopedic surgeons use cerclage cable, and this surgical technique is also worthy of promotion and application in clinical.

## Figures and Tables

**Table 1:** Comparison of basic data between the two groups.

Characteristics	PFNA+cable (*n* = 51)		PFNA (*n* = 69)	*t*/*χ*^2^	*p* value
Age (years)		83.0 ± 10.6	84.7 ± 7.5	1.029	0.306
BMI (kg/m^2^)		22.14 ± 2.52	22.93 ± 3.10	1.491	0.139
Gender	Men	13	18	5.451	0.941
	Women	38	51		
Preoperative diseases					
	Hypertension	23	38	1.167	0.280
	Diabetes	15	19	5.080	0.822
	Heart disease	12	23	1.364	0.243
	Others	15	12	2.430	0.119
ASA grading					
	II	23	37	0.853	0.356
	III	28	31	1.167	0.280
	IV	0	1	2.321	0.879
AO classification	31A2.2	23	30	3.120	0.860
	31A2.3	13	25	1.564	0.211
	31A3.1	4	6	2.790	0.867
	31A3.2	7	3	2.560	0.133
	31A3.3	4	5	5.192	0.820

**Table 2:** Surgical factors and follow-up results between the PFNA+cable group and the PFNA group.

Characteristics		PFNA+cable (*n* = 51)	PFNA (*n* = 69)	*t*	*p* value
Operation time (mins)		77.4 ± 10.6	73.5 ± 11.8	1.868	0.064
Intraoperative blood loss (ml)		191.4 ± 15.7	194.7 ± 13.2	1.249	0.214
Postoperative weight-bearing time (months)		2.94 ± 0.27	3.73 ± 0.71	7.546	0.0001
Fracture healing time (months)		3.36 ± 0.23	4.34 ± 0.22	23.66	0.0001
RUSH score	One month after operation	18.2 ± 0.8	14.8 ± 0.7	24.75	0.0001
	Three months after operation	25.7 ± 0.6	23.0 ± 1.1	15.86	0.0001
	Six months after operation	27.2 ± 0.9	25.1 ± 0.8	13.47	0.0001
	Twelve months after operation	28.5 ± 0.8	26.5 ± 0.6	15.65	0.0001
HHS	Preoperative	59.9 ± 7.3	60.7 ± 5.2	0.701	0.485
	One month after operation	76.7 ± 2.2	75.9 ± 2.8	1.812	0.075
	Three months after operation	86.2 ± 1.1	82.3 ± 1.6	14.98	0.0001
	Six months after operation	88.2 ± 0.8	83.5 ± 1.2	24.26	0.0001
	Twelve months after operation	96.4 ± 2.9	93.1 ± 3.2	5.809	0.0001
BI score	Preoperative	49.7 ± 5.3	48.9 ± 5.2	0.826	0.410
	One month after operation	54.2 ± 4.4	53.3 ± 3.7	1.215	0.227
	Three months after operation	83.8 ± 2.1	78.6 ± 2.6	11.73	0.0001
	Six months after operation	89.9 ± 0.7	84.6 ± 3.1	11.97	0.0001
	Twelve months after operation	95.0 ± 0.0	89.1 ± 2.5	16.83	0.0001

**Table 3:** HHS rating between the PFNA+cable group and the PFNA group at twelve months after operation.

Characteristics	PFNA+cable (*n* = 51)		PFNA (*n* = 69)	*χ* ^2^	*p* value
HHS rating				4.386	0.036
	80-89 good	2	11		
	90-100 excellent	49	58		

**Table 4:** Postoperative complications between the PFNA+cable group and the PFNA group.

Complications	PFNA	PFNA+cable
Superficial infection	2	1
Deep infection	3	2
VSD requirement	2	1
Nonunion	1	0
Screw penetration	2	0
Need for revision surgery	4	0
Screw cut-out	1	0

## Data Availability

Datasets analyzed during the current study are available from the corresponding author on reasonable request.
